# Supplementary material for: Reinterpretation of the results of randomized clinical trials
Source: PLoS One. 2024 Jun 14;19(6):e0305575. doi: 10.1371/journal.pone.0305575 (PMC11178203; doi:10.1371/journal.pone.0305575)
Supplement: S1 File — Data dictionary and important pieces of R codes. (PDF) [file pone.0305575.s001.pdf]

Supplementary materials

Data dictionary

id	RCT identification number
Name	RCT name
year	Year the RCT conducted
es	The observed effect size
se	The observed standard error
t	The computed Student's *t*
outcome	Type of RCT: DICH, dichotomous; CONT, continuous
n1	Sample size in the first arm
n2	Sample size in the second arm
C	The seriousness of type II relative to type I error
O	Prior odds of H_1 relative to H_0
pr	Probability corresponding to O
d	The effect size of interest
del	Delta (Eq. 8 in the article)
v	Degrees of freedom
t_opt	The optimum Student's *t*
p_opt	The optimum p value significance threshold
power_opt	The optimum study power
sig	If the RCT is "significant" based on p<0.05
sig2	If the RCT is "significant" based on p<0.005
Sig	If the RCT is "significant" based on optimum p approach
SIG	If the RCT is "significant" based on optimum p approach under the set constraints

Important pieces of the *R* codes

```
library("stats")
library("dplyr")
library("irr")

#-------- Functions --------------------------------
alpha <- function(t, v)
{
   return(2*pt(-abs(t), v, lower.tail=TRUE))
}

beta <- function(t, v, SNR)
{
   t = abs(t)
   return(pt(t - SNR, v, lower.tail=TRUE) - pt(-t - SNR, v, lower.tail=TRUE))
}

f_cost <- function(t, C, pr, v, SNR)
{
   return(C * pr * beta(t, v, SNR) + (1-pr) * alpha(t, v))
}

#---------------------------------------------------
```

```
#-------- Read data set ----------------------------
#--- Source: https://doi.org/10.17605/OSF.IO/XJV9G
#--- Part of the codes for filtering the data are kindly
#--- provided by Erik van Zwet.

set.seed(123)

load("CDSR.Rdata")
data %>%
  filter(RCT=="yes" & outcome.group == "efficacy" &
         (outcome.flag == "CONT" | outcome.flag == "DICH") &
          outcome.nr == 1 & abs(z)<20) %>%
  group_by(study.name) %>%
         sample_n(size = 1) -> dat #select single outcome per study

dat <- dat[dat$effect.N >= 10,]
dat$n1 <- as.numeric(regmatches(dat$effect.key, regexpr("\\d+$", dat$effect.key)))
dat$n2 <- dat$effect.N - dat$n1
dat %>%
  filter(n1 > 1 & n2 > 1) %>%
  select(id, study.name, study.year, n1, n2, effect.es, effect.se, effect.t,
         outcome.flag) -> dat

dat <- data.frame(dat)
colnames(dat) <- c("id", "Name", "year", "n1", "n2", "es", "se", "t", "outcome")

#-------- Set the parameters -----------------------
C <- 1/4
O <- 1/1
pr <- O/(O+1)

d <- ifelse(dat$n1 + dat$n2 < 100, 0.8, 0.5)
v <- dat$n1 + dat$n2 - 2
del <- d / sqrt(1/dat$n1 + 1/dat$n2)  #--  Assuming equal variance

#-------- Calculation of the optimum Student’s t and p significance threshold -----
dat$t_opt = rep(NA, nrow(dat))
for (i in 1:nrow(dat)){
   opt = optim(par = qt(1-0.05/2, v[i]), fn = f_cost, method = "L-BFGS-B",
               lower = 0, upper = Inf, C = C, pr = pr, v = v[i], SNR = del[i])
   dat$t_opt[i] = ifelse(opt$convergence == 0, opt$par, NA)
}
dat$p_opt <- alpha(dat$t_opt, v)
dat$power_opt <- 1 - beta(dat$t_opt, v, del)
dat$p <- alpha(dat$t, v)

dat$sig = ifelse(dat$p < 0.05, "Sig", "Not Sig")
dat$sig2 = ifelse((dat$p < 0.005), "Sig", "Not Sig")
dat$Sig = ifelse(dat$p < dat$p_opt, "Sig", "Not Sig")
dat$SIG = ifelse((dat$p_opt < 0.05) & (dat$power_opt >= 0.8) &
                 (dat$Sig == "Sig"), "Sig", "Not Sig")

#-------- Level of agreement -----------------------
kappa2(dat[, c("sig", "Sig")])
```
