# Supplementary material for: Reinterpretation of the results of randomized clinical trials
Source: PLoS One. 2024 Jun 14;19(6):e0305575. doi: 10.1371/journal.pone.0305575 (PMC11178203; doi:10.1371/journal.pone.0305575)
Supplement: Attachment — Submitted filename: PLOS-review-v02.1.docx [file pone.0305575.s002.docx]

The author chose C = .25 (line 133) based on the Handbook of Clinical Psychology. Is this justifiable when analyzing a broad range of RCTs that are mostly not related to psychology?

Line 88: The phrase ‘relative to H_0’ should be deleted.

Line 97: Instead of ‘the effect size of interest’, using ‘the minimum acceptable effect size’ is more accurate.

Line 106 Suggest to re-phrase as ‘R can be used’

Definition of C is inconsistent. Line 133 “*C*, the seriousness of type I error relative to type II error, was assumed to be 0.25” and Line 87 “*C* represents the relative seriousness of *β* compared to *α*”

Line 138: Suggest to re-phrase as ‘were looking for an effect size ≥ .5’

Line 140: Suggest to re-phrase as ‘were looking for an effect size ≥ .8’

Why does author choose *α* = .05 and *β* = .2 when calculating C-PST_opt.

A few graphs that show how PST_opt varies with C, pr, n1, n2 and the minimum acceptable effect size would be helpful.

Adding a table that displays values of PST_opt (assuming C = .25, pr = .5 and d = .5 or .8) at different n1 and n2 values would be useful to other researchers who might also want to apply the author’s method.

Line 204 ‘Fig 1’ should be ‘Fig 2’

Line 210: Suggest to re-phrase as ‘decreased by about 15 percentage points’

The author should mention in DISCUSSION the weaknesses of Eq. (1), the crux of his/her argument. Eq. (1) assumes all negatives have zero effect and all positives have an effect size that is equal to the minimum acceptable value. The reality is very different and certainly not binary. Another potential problem is that the author implicitly assumes the true size d of a positive effect depends on the cohort size of the study. This is of course incorrect. True effect size is an objective, worldly phenomenon regardless of how a trial is conducted.
