# Supplementary material for: Reinterpretation of the results of randomized clinical trials
Source: PLoS One. 2024 Jun 14;19(6):e0305575. doi: 10.1371/journal.pone.0305575 (PMC11178203; doi:10.1371/journal.pone.0305575)
Supplement: Attachment — Submitted filename: Review Comments_0425.docx [file pone.0305575.s003.docx]

**Review Comments**

**Summary**: This paper proposes an approach selecting appropriate cut-off values for a statistical test and applies it to reanalyze a collection of clinical trials. While it provides some kind of guidance on selecting significance cut-off thresholds, the paper is not well-written, and I do not recognize the necessity of publishing it.

**Major comments**:

1. This paper lacks a literature review on the past developments regarding the selection of significance cut-off thresholds for a statistical test. There is not a clear justification provided for using the proposed error function as an objective. Additionally, the components of the objective function are not introduced clearly. For example, while the objective function is a weighted sum of type-I error and type-II error, the weights are not well-defined or interpreted. The objective uses the information on type-II error (or equivalently, power), yet the power is seldom mentioned in the abstract and introduction. It is introduced alongside the objective error function without proper explanations.
2. The statistical methods section lacks organization. For instance, it would be better to omit the expressions for the distribution function of $t$-distribution and instead provide explanations for $\delta$ before giving the distribution function. In addition, the author fails to introduce statistical assumptions necessary for performing the calculations. For example, it is implicitly assumed that two groups are independent and normally distributed with the same variance. I do not think the current level of rigor is sufficient for introducing a completely new approach without acknowledging previous developments. In addition, using $t$-test only may not be always appropriate.
3. The author employs overly definitive language, which appears very weak to me. For example, the author states, ‘Frequentist statistics has internal conflict. …’ (line 31-33), yet little evidence is given to support why the Bayesian statistics is better than the frequentist statistics, even in this specific question. I strongly disagree with making such assertions without giving sound evidence because the methods proposed in this paper do not use any knowledge or setup from Bayesian’s framework.
4. This paper only proposes a new method without giving any discussions on the limitations or drawbacks. For example, using the proposed objective function depends on tuning multiple parameters and minimizing an objective involving the power may not be the best way of controlling false-positive.
5. There may be many other interesting aspects relevant to the research question in this paper. For example, the impact of choosing weights on the resulting optimal cut-off values, and the relationship between parameters and final optimizer (e.g., monotone or quadratic). Why the objective is a weighted sum instead of other convex shapes? The current statistics is $t$-test and what is the consequence of using other test statistics?

**Minor comments**:

1. In the background of the abstract, the author states, ‘Many researchers show that results of RCTs are mostly false-positive’ (line 15-16). In my opinion, a false-positive is defined as the event incorrectly rejecting the null hypothesis. One cannot identify a false-positive without knowing beforehand that the null hypothesis is true. Statistically speaking, a false-positive is a binary random variable with a prespecified probability $\alpha$, known as type-I error rate, which is a crucial quantity that a statistician would like to control.
2. In the discussion section (line 231-244), the author argues for viewing the parameters as a posteriori instead of priori. I think there is gap between current methods and methods capable of handling parameters as a posterior. I suggest filling in this blank in the methods section.
3. In the discussion section (line 255-258), the author mentions that the computational intensity may be a drawback when using the Bayesian’s framework. However, in my opinion, many modern computers can already handle the computational task if the model is not overly complex, e.g., the levels of the data are not too large.
4. This paper lacks a systematic simulation study to demonstrate the control of false-positives and false-negatives.
